# New-onset dermatomyositis following SARS-CoV-2 infection and vaccination: a case-based review

**DOI:** 10.1007/s00296-022-05176-3

**Published:** 2022-08-08

**Authors:** Marie-Therese Holzer, Martin Krusche, Nikolas Ruffer, Heinrich Haberstock, Marlene Stephan, Tobias B. Huber, Ina Kötter

**Affiliations:** 1 III. Department of Medicine, University Medical Center Hamburg-Eppendorf, Hamburg, Germany; 2 Department of Rheumatology and Immunology, Klinikum Bad Bramstedt, Bad Bramstedt, Germany

**Keywords:** Dermatomyositis, COVID-19, COVID-19 vaccines, SARS-CoV-2

## Abstract

Dermatomyositis is a rare, type I interferon-driven autoimmune disease, which can affect muscle, skin and internal organs (especially the pulmonary system). In 2021, we have noted an increase in new-onset dermatomyositis compared to the years before the SARS-CoV-2 pandemic in our center. We present four cases of new-onset NXP2 and/or MDA5 positive dermatomyositis shortly after SARS-CoV-2 infection or vaccination. Three cases occurred within days after vaccination with Comirnaty and one case after SARS-CoV-2 infection. All patients required intensive immunosuppressive treatment. MDA5 antibodies could be detected in three patients and NXP2 antibodies were found in two patients (one patient was positive for both antibodies). In this case-based systematic review, we further analyze and discuss the literature on SARS-CoV-2 and associated dermatomyositis. In the literature, sixteen reports (with a total of seventeen patients) of new-onset dermatomyositis in association with a SARS-CoV-2 infection or vaccination were identified. Ten cases occurred after infection and seven after vaccination. All vaccination-associated cases were seen in mRNA vaccines. The reported antibodies included for instance MDA5, NXP2, Mi-2 and TIF1γ. The reviewed literature and our cases suggest that SARS-CoV-2 infection and vaccination may be considered as a potential trigger of interferon-pathway. Consequently, this might serve as a stimulus for the production of dermatomyositis-specific autoantibodies like MDA5 and NXP2 which are closely related to viral defense or viral RNA interaction supporting the concept of infection and vaccination associated dermatomyositis.

## Introduction

Dermatomyositis is a rare disease with an incidence of 1 to 15 per million [1]. Apart from muscle and skin, the disease can also affect other organs, such as lungs, heart, and blood vessels with varying clinical outcomes, depending on the specific antibody [2]. Although the pathophysiology has not yet been fully elucidated, type I interferon (IFN) is now known to play a key role in the development of the disease. Induction of interferon-stimulated genes can be seen in muscle biopsies of dermatomyositis and type I IFN signature has been reported in peripheral blood samples [3, 4]. Specifically, anti-melanoma differentiation-associated protein 5 (anti-MDA5) antibody-positive dermatomyositis patients showed very high serum type I IFN signature [5].

Interestingly, MDA5 positive dermatomyositis and SARS-CoV-2 infection share clinical and laboratory features, such as inflammatory cytokine profile and interstitial lung involvement [6]. Furthermore, creatine kinase (CK) elevation has been reported in up to 27% of SARS-CoV-2-infected patients [7]. Inflammatory myopathy has been detected in infected patients as well as autoantibody production against nuclear matrix protein-2 (NXP2) and MDA5 without clinical symptoms of dermatomyositis but a correlation of worse pulmonary outcomes [8, 9].

The newly developed messenger ribonucleic acid (mRNA) vaccine is known to induce an IFN signaling, partly also via MDA5 [10]. After SARS-CoV-2 vaccination, elevated IFN levels can be detected in healthy individuals [11]. So far, the development of autoimmune diseases like systemic lupus erythematosus (SLE) [12] and autoimmune myositis [13] after SARS-CoV-2 vaccination have been reported in a few case reports.

Both, SARS-CoV-2 infection and vaccination, may lead to new-onset dermatomyositis via autoimmunity due to interferon signaling, hyperinflammation and autoantibody induction.

## Case presentation

**Fig. 1:**
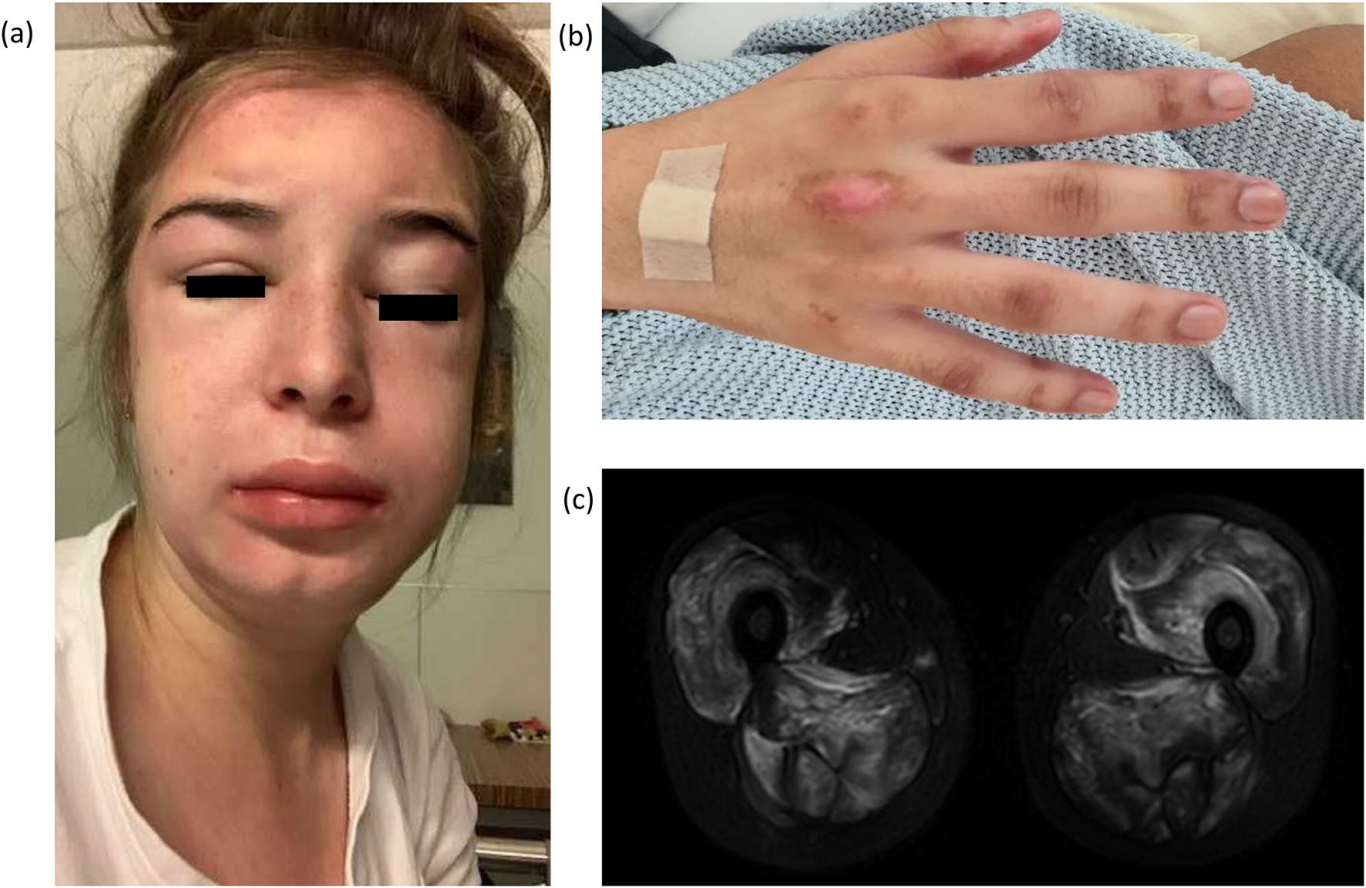
Patients’ images: **a** Patient 2: facial swelling, heliotrope erythema. **b** Patient 1: Gottron papules **c** Patient 2: magnetic resonance imaging scan (T2) showing bilateral active myositis in the adductors and extensors of the thighs

We report four cases with the occurrence of MDA5 and/or NXP2 positive dermatomyositis directly linked to SARS-CoV-2 infection or vaccination. Our sample comprises three female and one male patient ranging from 19 to 57 years of age. Three patients experienced the onset of dermatomyositis shortly after SARS-CoV-2 mRNA vaccination with BNT162b2 (Comirnaty) (1–7 days) and one patient 2 weeks after SARS-CoV-2 infection. Intriguingly, patient 1 developed dermatomyositis after his first vaccination, whereas dermatomyositis in patients 3 and 4 evolved after the second vaccination. All patients showed typical skin manifestations and reported proximal myalgia (Fig. 1). Two patients initially presented with arthritis. One patient had severe dyspnea, and another had excessive dysphagia. Only two patients had elevated CK levels. MDA5 antibodies could be detected in three patients and NXP2 antibodies were found in two patients (patient 3 was positive for both antibodies). In three patients, muscle magnetic resonance imaging was performed, showing bilateral proximal myositis. Patient 1, furthermore, developed rapid-progressive interstitial lung disease (RP-ILD). Skin and muscle biopsies showed pathologies consistent with dermatomyositis.

All patients required immunosuppression and were treated with glucocorticoid pulse therapy. Whilst patients 3 and 4 showed mild symptoms that were successfully treated with hydroxychloroquine and azathioprine; patients 1 and 2 had a long hospitalization with multiple intensive care treatments due to life-threatening major organ involvements. Both patients required extensive immunosuppression including ciclosporin A, mycophenolate mofetil and rituximab. Table 1 displays patients’ characteristics and therapeutic concepts.

**Table 1:** Patients’ characteristics

	Patient 1	Patient 2	Patient 3	Patient 4
Age (years)	19	20	57	51
Sex	Male	Female	Female	Female
Symptom onset	5 days after 1st vaccination with BNT162b2 (Comirnaty)	2 weeks after infection	1 week after 2nd vaccination with BNT162b2 (Comirnaty)	1 day after 2nd vaccination with BNT162b2 (Comirnaty)
Skin manifestation	Gottron papules and Gottron signs over extensor sides of elbows and knees, Hiker’s feet	Heliotrope erythema, Gottron papules, scalp exanthem, V and Shawl sign, facial swelling	Heliotrope erythema, Gottron papules, Gottron signs at the elbows, erythematous macular rash on forehead, Shawl sign, periungual erythematous swelling	Reddened painful fingertips (Chillblain lesions) and periungual erythematous swelling, Gottron papules, heliotrope rash and occipital lesions
Organ involvement	Proximal myalgia, arthritis, RP-ILD	Proximal myalgia (including extensive dysphagia)	Proximal myalgia	Proximal myalgia, arthritis
Muscle MRI findings	Bilateral myositis of muscles inserting trochanter minor and major	Bilateral myositis of muscles of the pelvic hip girdle and thighs	Bilateral myositis of muscles of the shoulders and thighs	No MRI performed
Antinuclear antibody	< 1:80	1:640	1:2560	1:5120
Myositis specific antibodies	MDA5, RO-52	NXP2	MDA5, NXP2	MDA5
CK (U/l) (normal < 190)	1074	19,647	146	66
LDH (U/l) (normal 120–250)	839	1903	215	125
CRP (mg/l) (normal < 5)	< 5	< 5	< 5	< 5
Biopsies	Muscle: mild myopathy and increased MHC I expression Skin: perivascular neutrophilic infiltrates	Muscle: necrosis, expression of MAC and MHC I Skin: interface-dermatitis and perivascular lymphocytic dermatitis	No biopsy performed	Skin: perivascular lymphocytic infiltrates consistent with DM
Treatment	Glucocorticoids, IVIG, Tofacitinib, MMF, Rituximab, Ciclosporin A, Anakinra, Nintedanib, Daratumumab	Glucocorticoids, IVIG, MMF, Ciclosporin A, Tofacitinib, Rituximab	Glucocorticoids, Hydroxychloroquine, Azathioprine	Glucocorticoids, MTX s.c., Hydroxychloroquine, Azathioprine

*RP-ILD* Rapidly progressive interstitial lung disease, *MRI* magnetic resonance imaging, *MDA5* Melanoma differentiation-associated protein 5, *NXP2* Nuclear matrix protein 2, *CK* Creatine kinase, *LDH* Lactate dehydrogenase, *AST* Aspartate aminotransferase, *CRP* C-reactive protein, *MHC I* Major histocompatibility complex, *MAC* Membrane attack complex, *DM* Dermatomyositis, *IVIG* Intravenous immunoglobulin, *MMF* Mycophenolate Mofetil, *MTX* Methotrexate

Moreover, we have noted an increase of dermatomyositis diagnoses in our center since the beginning of the SARS-CoV-2 pandemic with almost a doubling of new-onset dermatomyositis in overall inpatient cases from 0.06 to 0.15% (2017–2020) up to 0.26% in the year 2021 (Table 2).

**Table 2:** Comparison of new-onset dermatomyositis (DM) over the last 5 years

Year	New-onset DM cases^a^	Autoantibodies	Total number of inpatients	Percentage^b^
2017	2	NXP2, Ro52 Mi2	1671	0.12%
2018	2	MDA5 Antibody negative	1342	0.15%
2019	1	Mi2	1207	0.08%
2020	1	Mi2, TIF1γ	1720	0.06%
2021	5	NXP2 MDA5, Ro52 MDA5 NXP2, MDA5 Antibody negative	1895	0.26%

^a^paraneoplastic associated DM excluded

^b^percentage = (new-onset DM case) ÷ (total number of inpatients)

## Methods

**Fig. 2:**
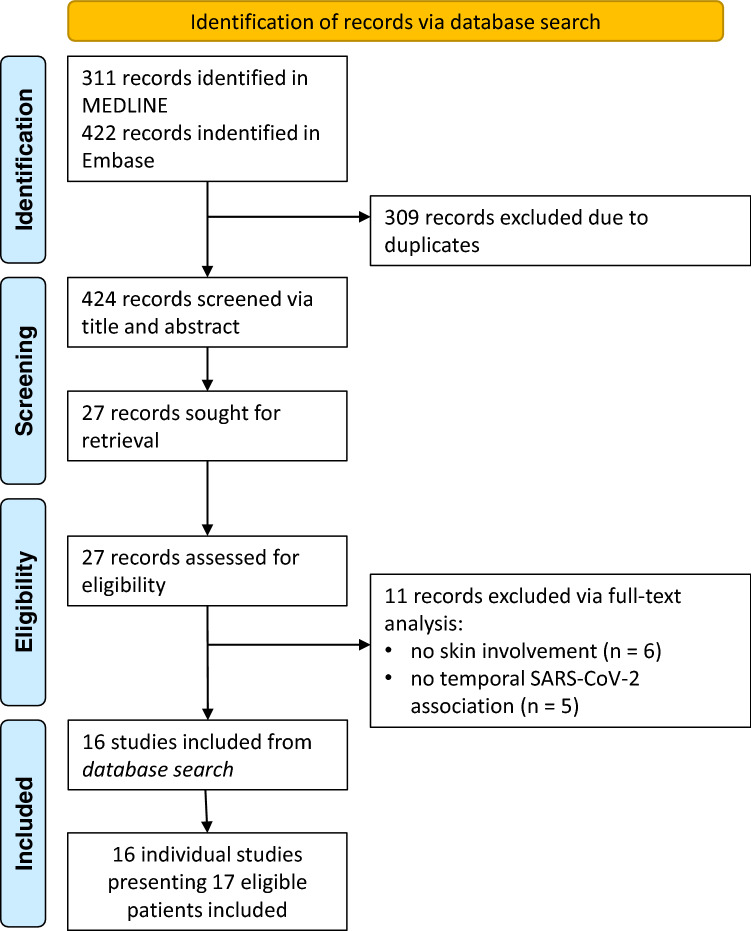
Methodology flowchart of systematic literature review. *n* number

To identify previously reported cases of SARS-CoV-2 associated dermatomyositis, a systematic review of the literature according to PRISMA guidelines was performed. MEDLINE and Embase were systematically searched until the 25^th^ of May 2022. The search strategy included the following terms to identify dermatomyositis cases: ‘myositis’, ‘dermatomyositis’, ‘polymyositis’, ‘rhabdomyolysis’, ‘antisynthetase syndrome’ and ‘inflammatory myopathy’. SARS-CoV-2 association was established with ‘SARS-CoV-2’, ‘COVID-19’ and ‘coronavirus’. All terms were used to search titles and abstracts of publications. The search was conducted as (‘myositis’ OR ‘dermatomyositis’ OR ‘polymyositis’ OR ‘rhabdomyolysis’ OR ‘antisynthetase syndrome’) AND (‘SARS-CoV-2’ OR ‘COVID-19’ OR ‘coronavirus’). The database search in MEDLINE identified 311 publications, the database search in Embase 422, which were independently reviewed by two authors (MTH, NR). A third independent reviewer (MK) decided in case of discrepancy. Based on the EULAR/ACR criteria for (juvenile) dermatomyositis [14], new-onset cases of dermatomyositis with a temporal relation to SARS-CoV-2 infection or vaccination were included in this review. Non-English articles, reviews without description of detailed case information and congress abstracts were excluded. Finally, 16 studies reporting 17 cases were included. The methodology flowchart is shown in Fig. 2.

## Results

The clinical, laboratory, radiographic and histopathologic features of SARS-CoV-2 infection-/vaccination-associated dermatomyositis of the identified 17 cases of the systematic review are summarized in Tables 3 and 4 [13, 15–29]. Interestingly, 70.6% of the patients were female, mean age was 52.4 years. Ten cases occurred after infection and seven after vaccination. All reported vaccinations were mRNA vaccination. Six of these seven cases were after BNT162b2 (Comirnaty) and one after mRNA-1273 (Spikevax) vaccination. All identified cases had pathognomonic skin manifestations. Myocardial involvement was assumed in two cases (one after infection and one after Comirnaty vaccination). Lung involvement was reported in seven patients. Five of these lung involvements were reported after SARS-CoV-2 infection. One patient with MDA5, and two patients with NXP2-antibodies were reported. Furthermore, four Mi-2 positive patients, two RNP/TIF1γ, respectively, and one Jo-1 positive patient were identified. All patients received glucocorticoids and nine patients IVIG. One patient had a lethal disease course.

**Table 3:** Clinical, laboratory, radiologic and histopathologic features of SARS-CoV-2 infection/vaccination associated dermatomyositis cases found in systematic search [13, 15–29]^a^

Author, year	Patient’s age in years, sex	Infection/ 1st, 2nd vaccination (with)	Myositis-specific antibodies	Creatine kinase	Muscle biopsy	MRI	Extramuscular involvement	Treatment	Outcome
Borges et al., 2021	36, Female	Infection	Mi-2	3518 U/l	Not performed	Not performed	Skin	GC	Improvement
Camargo Coronel et al. 2022	76, Female	2nd vaccination (BNT162b2, Comirnaty)	Mi-2	3368 U/l	Consistent with DM	Not performed	Skin, dysphagia	GC, MTX	Improvement
Derbel et al. 2021	61, Female	Infection	Jo-1	1052 U/l	Not performed	Not performed	Skin, possibly lung, joints	GC	Improvement
Gokhale et al. 2020	64, Male	Infection	Negative	990 U/l	Not performed	Positive	Skin, possibly lung, dysphagia	GC, IVIG, MMF	Improvement
Gokhale et al. 2020	50, Male	Infection	Mi-2	1169 U/l	Not performed	Positive	Skin, possibly lung	GC, IVIG, MTX	Improvement
Gouda et al. 2022	43, Female	2nd Vaccination (BNT162b2, Comirnaty)	RNP	3358 µg/l	Not performed	Positive	Skin, lung, joints	GC, MMF, HCQ	Improvement
Ho et al. 2021	58, Male	Infection	Negative	9684 U/l	Consistent with DM	Not performed	Skin, possibly lung	GC, MTX	Improvement
Keshtkarjahromie et al. 2021	65, Female	Infection	MDA5	1222 U/l	Not performed	Positive	Skin, possibly lung, joints	GC, IVIG	Death
Kreuter et al. 2022	68, Female	2nd Vaccination (BNT162b2, Comirnaty)	TIF1γ	Not stated	Not performed	Not performed	Skin	GC	Improvement
Lee et al. 2022	53, Male	2nd vaccination (BNT162b2, Comirnaty)	NXP2	14,659 U/l	Consistent with DM	Positive	Skin, dysphagia	GC, IVIG, RTX	Improvement
Liquidano-Perez et al. 2021	4, Female	Infection	RNP	403 mg/dl	Not performed	Positive	Skin, possibly lung, dysphagia	GC, IVIG, MTX, CsA	Improvement
Okada et al. 2021	64, Female	Infection	NXP2	1495 U/l	Consistent with DM	Positive	Skin	GC, AZA	Improvement
Rodero et al. 2022	15, Female	Infection	Negative	545 U/l	Consistent with DM	Not performed	Skin	GC, IVIG, Tofacitinib	Improvement
Shahidi Dadras et al. 2021	58, Female	Infection	Negative	2611 U/l	Not performed	Not performed	Skin, myocardial involvement	GC, MTX, HCQ	Improvement
Venkateswaran et al. 2022	43, Male	1st Vaccination (mRNA-1273, Spikevax)	Negative	Not stated	Not performed	Not performed	Skin	GC, IVIG	Improvement
Vutipongsatorn et al. 2022	55, Female	1st Vaccination (BNT162b2, Comirnaty)	Mi-2	11,330 U/l	Not performed	Positive	Skin, myocardial involvement	GC, IVIG, CYC	Improvement
Wu et al. 2022	77, Female	1st Vaccination (BNT162b2, Comirnaty)	TIF1γ	4476 U/l	Consistent with DM	Not performed	Skin	GC, IVIG	Improvement

*MRI* Magnetic resonance imaging, *DM* Dermatomyositis, GC: Glucocorticoids, *MTX* Methotrexate, *IVIG* Intravenous immunoglobulin, *MMF* Mycophenolate Mofetil, *RNP* Ribonucleoprotein, *TIF1γ* Transcription intermediary factor 1γ, *MDA5* Melanoma differentiation-associated protein 5, *NXP2* Nuclear matrix protein 2, *RTX* Rituximab, *CsA* Ciclosporin A, *AZA* Azathioprine, *HCQ* Hydroxychloroquine, *CYC* Cyclophosphamide

^a^alphabetically ordered

**Table 4:** Analysis of clinical, laboratory, radiologic and histopathologic features of SARS-CoV-2 infection/vaccination associated dermatomyositis cases found in the systematic review [13, 15–29]

		Total	Percentage
Sex	Male	5	29.4%
	Female	12	70.6%
Age (years)	Mean	52.4	–
	Median	58.0	–
Infection	Negative	7	41.2%
	Positive	10	58.8%
Vaccination	Negative	10	58.8%
	Positive	7	41.2%
Vaccine	BNT162b2 (Comirnaty)	6	85.7%
	mRNA-1273 (Spikevax)	1	14.3%
	First vaccine	3	42.9%
	Second vaccine	4	57.1%
MSA	MDA5	1	5.9%
	NXP2	2	11.8%
	Mi-2	4	23.5%
	RNP	2	11.8%
	TIF1γ	2	11.8%
	Jo-1	1	5.9%
	Negative	5	29.4%
Creatine kinase (U/l)	Mean	3230	–
	Median	2053	–
Muscle biopsy	Not performed	11	64.7%
	Performed	6	35.3%
	Consistent with myositis	5	29.4%
MRI	Not performed	9	52.9%
	Performed	8	47.1%
	Consistent with myositis	8	47.1%
Skin	Negative	0	0.0%
	Positive	17	100.0%
	Not reported	0	0.0%
Lung	Negative	6	35.3%
	Positive	7	41.2%
	Possible SARS-CoV-2 manifestation	5	29.4%
	Not reported	4	23.5%
Myocardial involvement	Negative	1	5.9%
	Positive	2	11.8%
	Not reported	14	82.4%
Dysphagia	Negative	0	0.0%
	Positive	4	23.5%
	Not reported	13	76.5%
Arthritis	Negative	0	0.0%
	Positive	3	17.6%
	Not reported	14	82.4%
Glucocorticoids	Negative	0	0.0%
	Positive	17	100.0%
	Not reported	0	0.0%
IVIG	Negative	0	0.0%
	Positive	9	52.9%
	Not reported	8	47.1%
CYC	Negative	0	0.0%
	Positive	1	5.9%
	Not reported	16	94.1%
RTX	Negative	0	0.0%
	Positive	1	5.9%
	Not reported	16	94.1%
MMF	Negative	0	0.0%
	Positive	5	29.4%
	Not reported	12	70.6%
Other treatment	Cyclosporine	1	5.9%
	Azathioprine	1	5.9%
	Tofacitinib	1	5.9%
	Hydroxychloroquine	2	11.8%
Outcome	Death	1	5.9%
	Clinical improvement	16	94.1%

*MSA* Myositis-specific antibodies, *MDA5* Melanoma differentiation-associated protein 5, *NXP2* Nuclear matrix protein 2, *RNP* Ribonucleoprotein, *TIF1γ* Transcription intermediary factor 1γ, *MRI* Magnetic resonance imaging, *IVIG* Intravenous immunoglobulin, *CYC* Cyclophosphamide, *RTX* Rituximab, *MMF* Mycophenolate Mofetil

## Discussion

The reported cases vary in autoimmune serology, clinical course, and prognosis. Nevertheless, the common feature was the new-onset dermatomyositis shortly after SARS-CoV-2 infection or vaccination.

Interestingly, lung involvement was the most frequent manifestation (despite skin and muscle). We would like to highlight, that after SARS-CoV-2 infection, radiographically changes of the lung might sometimes be hard to differentiate between infection- or autoimmune-disease related.

In general, viral infections are a well-known trigger of dermatomyositis [30]. Furthermore, seasonal clustering of MDA5-positive dermatomyositis with lower incidence in European summer months is known [31].

In the systematic database search, we identified ten cases of new-onset dermatomyositis after SARS-CoV-2 infection and one patient in our cohort.

In some of these cases apart from classical clinical and laboratory findings of dermatomyositis an IFN signature as well as autoinflammatory clinical aspects have been reported [15, 17, 26].

Consistent with the results of our center, Gokhale et al. also reported an increase of new-onset dermatomyositis in a center in Mumbai with five new cases of dermatomyositis in 6 months from April 2020 (usually one to two new cases per year) [20]. Furthermore, Movahedi et al. described an increase of new-onset juvenile dermatomyositis in Iran. Regularly, two to four new cases were admitted each year from the years 2014 to 2019, whereas from February 2020 to February 2021 eight new-onset juvenile dermatomyositis cases were registered [32].

MDA5- and NXP2-antibodies were reported in each four of the 21 identified cases (16.7%, respectively). Both antibodies are associated with viral interaction in general: MDA5 is an intracellular sensor for viral RNA, triggering proinflammatory immune response especially involving type I IFN [32]. NXP2 shows RNA binding activity and upregulation of its expression has been detected in influenza infection [33]. Furthermore, the two antibodies have been associated with SARS-CoV-2-infections: In a small study of 35 SARS-CoV-2 patients, de Santis et al. reported the occurrence of NXP2 (*n* = 3) and MDA5 antibodies (*n* = 1). Both antibodies were associated with a severe disease course [9]. In SARS-CoV-2 infection, MDA5 was shown to guide an innate immune response via IFN signaling [34]. It has been hypothesized, that viral RNA may trigger MDA5 expression and cell damage may lead to MDA5 release followed by autoantibody production [35]. In addition, Wang et al. demonstrated correlative evidence between high titer of anti-MDA5 antibodies and lethal outcome of SARS-CoV-2 infection. Of the 274 patients analyzed, 48.2% were anti-MDA5 positive and high antibody titer (> 10 U/ml) was more frequent in non-survivors [36].

In addition, muscle involvement seems to be an important feature of SARS-CoV-2 infection. Elevated CK was detected in 27% of the SARS-CoV-2 patients [7]. Furthermore, inflammatory myopathy was seen in SARS-CoV-2 patients without significant signs of viral infection of myocytes suggesting autoimmune features [8]. In addition, Manzano et al. discovered the presence of myxovirus resistance protein A (MxA), a type I IFN induced protein, in the muscle biopsy of an SARS-CoV-2 patient with proximal myopathy, suggesting parts of the inflammatory myopathy caused by interferonopathy [38]. Another study also showed immune-mediated and inflammatory myopathy in 16 of 35 autopsies of deceased SARS-CoV-2 patients with high expression of major histocompatibility complex (MHC) I and MxA expression in some cases, which was not seen in controls [39], underlining a possible IFN and cytokine triggered mechanism. These MHC I and IFN patterns found in muscles of SARS-CoV-2 patients closely resemble the pattern found in muscle biopsies in dermatomyositis [2].

Furthermore, the development of autoimmune diseases after vaccination by molecular mimicry and bystander activation in genetically susceptible individuals has frequently been discussed [40, 41]. There have been also a few case reports of vaccinations as a potential trigger of dermatomyositis but no significant association has been established in previous vaccination studies [42].

Rare, but possible side effects after SARS-CoV-2 vaccination, such as the development of autoimmune diseases such as systemic lupus erythematosus (SLE), myocarditis, vasculitis, and thrombotic thrombocytopenia have been reported [12, 43–46]. In the last few months, since the beginning of the global vaccination campaign, apart from the mentioned autoimmune diseases after vaccination, myositis following SARS-CoV-2 vaccination has been reported. In the reviewed literature and our cohort, we detected ten patients with new-onset dermatomyositis after SARS-CoV-2 vaccination. All patients received a mRNA vaccination. Interestingly, six patients developed the disease after the second vaccination. Whilst mRNA vaccination seems to be more prevalent for dermatomyositis-association, other autoimmune diseases like thrombotic thrombocytopenia or SLE seem more likely to occur after adenovirus vector vaccine like ChAdOx1-S. Autoantibody production and activation is discussed as possible mechanism [12]. Furthermore, there are reports of myositis in temporal association to ChAdOx1-S vaccination [37].

In SARS-CoV-2 mRNA vaccines, the mRNA enters human cells via angiotensin-converting enzyme 2 and induces an immune response to develop spike antibodies against SARS-CoV-2 infection and memory T and B cells [47]. During the development of mRNA vaccine, a strong type I IFN response with MDA5 as one of the possible RNA sensing and IFN inducing mechanisms was seen [10]. Thus, the nowadays used mRNA vaccines are containing nucleoside-modified mRNA, which reduces the IFN pathway activation [10, 48]. Nevertheless, increased type I IFN levels were detected after mRNA vaccination against SARS-CoV-2, but they were comparable to IFN levels after influenza vaccination [11]. As dermatomyositis is known to be an IFN driven disease, there might be a tipping point inducing autoimmunity due to the vaccination response in some patients.

Most recently, Yin et al. were able to prove the importance of the NLRP3 inflammasome in the pathophysiology of dermatomyositis [49]. NLRP3 inflammasome activation has also been detected in myocarditis after mRNA vaccination. It is assumed, that similarly to SARS-CoV-2 infection, spike protein might trigger NLRP3 inflammasome activity, or that the lipid nanoparticles used, might stimulate the NLRP3 inflammasome [50, 51]. This might present another additional pathomechanism in the development of autoimmune diseases like dermatomyositis following mRNA vaccination.

In summary, this case series and the reviewed literature suggest an association between SARS-CoV-2 infection/vaccination and the development of dermatomyositis, since all reported cases occurred within a very short timeframe after vaccination or infection. Possible pathophysiological mechanisms may include type I IFN pathways, the NLRP3 inflammasome and the induction of autoantibody production (especially of those antibodies, which are closely related to viral defense or viral RNA interaction like MDA5 and NXP2).

Due to the limited number of identified cases, we would like to emphasize that the association between SARS-CoV-2 infection/vaccination and the development of dermatomyositis does not necessarily prove causality, and further research is needed.

We would like to underline that the benefit of SARS-CoV-2 vaccinations highly outweighs possible very rare autoimmune phenomena. Nevertheless, rheumatologists should be aware of possible associations between dermatomyositis and SARS-CoV-2 infection/vaccination to maintain optimal medical management.

## Data Availability

The data underlying this article cannot be shared publicly due to the anonymization of patients and for the privacy of individuals that participated in this case series. Non-confidential data will be shared on reasonable request to the corresponding author.
